# Exercise Limitation after Critical Versus Mild COVID-19 Infection: A Metabolic Perspective

**DOI:** 10.3390/jcm11154322

**Published:** 2022-07-25

**Authors:** Maurice Joris, Joël Pincemail, Camille Colson, Jean Joris, Doriane Calmes, Etienne Cavalier, Benoit Misset, Julien Guiot, Grégory Minguet, Anne-Françoise Rousseau

**Affiliations:** 1 Department of Sports Medicine, Malvoz Institute, 4020 Liège, Belgium; cms.joris@skynet.be; 2 Clinical Chemistry Department, University Hospital, University of Liège, 4000 Liège, Belgium; j.pincemail@chuliege.be (J.P.); etienne.cavalier@chuliege.be (E.C.); 3 Department of Intensive Care and Burn Center, University Hospital, University of Liège, 4000 Liège, Belgium; camille.colson@chuliege.be (C.C.); benoit.misset@chuliege.be (B.M.); 4 Department of Anaesthesiology, University Hospital, University of Liège, 4000 Liège, Belgium; jean.joris@chuliege.be (J.J.); gminguet@chuliege.be (G.M.); 5 Department of Pneumology, University Hospital, University of Liège, 4000 Liège, Belgium; dcalmes@chuliege.be (D.C.); jguiot@chuliege.be (J.G.)

**Keywords:** cardiopulmonary exercise testing, COVID-19, critical illness, survivors, long COVID, obesity, hypermetabolism, oxidative stress, inflammation

## Abstract

Exercise limitation in COVID-19 survivors is poorly explained. In this retrospective study, cardiopulmonary exercise testing (CPET) was coupled with an oxidative stress assessment in COVID-19 critically ill survivors (ICU group). Thirty-one patients were included in this group. At rest, their oxygen uptake (VO_2_) was elevated (8 [5.6–9.7] mL/min/kg). The maximum effort was reached at low values of workload and VO_2_ (66 [40.9–79.2]% and 74.5 [62.6–102.8]% of the respective predicted values). The ventilatory equivalent for carbon dioxide remained within normal ranges. Their metabolic efficiency was low: 15.2 [12.9–17.8]%. The 50% decrease in VO_2_ after maximum effort was delayed, at 130 [120–170] s, with a still-high respiratory exchange ratio (1.13 [1–1.2]). The blood myeloperoxidase was elevated (92 [75.5–106.5] ng/mL), and the OSS was altered. The CPET profile of the ICU group was compared with long COVID patients after mid-disease (MLC group) and obese patients (OB group). The MLC patients (*n* = 23) reached peak workload and predicted VO_2_ values, but their resting VO_2_, metabolic efficiency, and recovery profiles were similar to the ICU group to a lesser extent. In the OB group (*n* = 15), no hypermetabolism at rest was observed. In conclusion, the exercise limitation after a critical COVID-19 bout resulted from an altered metabolic profile in the context of persistent inflammation and oxidative stress. Altered exercise and metabolic profiles were also observed in the MLC group. The contribution of obesity on the physiopathology of exercise limitation after a critical bout of COVID-19 did not seem relevant.

## 1. Introduction

Post-acute COVID-19, also named long COVID, is a syndrome characterized by persistent symptoms and/or delayed or long-term complications lasting more than several weeks from the onset of the disease [1,2]. The symptoms can affect patients after all levels of disease severity, even when not requiring hospitalization. Long COVID-19 prevalence depends on many factors, including the studied region, follow-up time, and studied symptoms. This prevalence has been estimated to be around 30% in patients who did not need hospitalization during an acute infection [3]. In patients who survived a COVID-19-related acute respiratory distress syndrome (ARDS) with ICU stay, long COVID may overlap with the post-intensive care syndrome [4]. Patients with long COVID syndrome complain of muscle dysfunction with fatigue, muscle weakness, and decline in functional performances [5].

Using cardiopulmonary exercise testing (CPET), the most objective and integrative exercise-capacity assessment technique [6], exercise limitation has been confirmed in a few small reports in long COVID patients even after mild infection [7,8]. Dysfunctional breathing with hyperventilation was described in this context [8,9,10]. However, these findings remain poorly explained and investigated. In a recent study, we confirmed a marked exercise limitation in critically ill COVID-19 survivors 3 months after intensive care unit (ICU) discharge, mainly related to metabolic disorders rather than cardiac or pulmonary residual impairments. The exercise limitation persisted 6 months after ICU discharge [11]. Of note, all these patients were obese, as an overweight condition and obesity were observed in most patients with severe COVID-19 [12,13,14]. However, the role of obesity in the observed exercise intolerance in these critically ill COVID-19 survivors is unknown. Yet, obesity was associated with chronic low-grade inflammation [15,16], which is a potential trigger for metabolic disorders after critical illness [17,18].

The primary aim of this retrospective study was to correlate exercise capacity using CPET with an oxidative stress assessment in critical COVID-19 survivors 3 months after discharge. The secondary aim was to compare their exercise capacity to those of patients with long COVID after a mild infection and to those of patients with obesity.

## 2. Materials and Methods

### 2.1. Patients and Data Sources

This retrospective study was conducted at the University Hospital of Liège and in the sports medicine unit of the Province of Liège. The ethics committee of the University Hospital of Liège (Chair Prof V. Seutin, Liège, Belgium) reviewed the study and approved it (local reference: 2022/77, 5 April 2022). Informed consent was not required because the study was retrospective.

#### 2.1.1. COVID-19 ARDS Survivors (ICU Group)

In our university hospital, patients surviving an ICU stay ≥7 days are routinely invited to our post-intensive care follow-up clinic at one, three, six, and twelve months after ICU discharge. Each visit is held by a multidisciplinary team, including a critical care physician, a critical care nurse, a physiotherapist, and a dietician. The examination content is standardized, addressing the patient’s physical status and functional performances, mental health disorders, cognitive impairment, sleep disorders, and health-related quality of life. Patients do not enter the post-ICU trajectory of our follow-up clinic if they are unable to communicate in French, the local language; if they have been transferred to another hospital; or if we are unable to give them information about the post-ICU follow-up clinic. The scheduled face-to-face consultation is generally cancelled if they are still hospitalized in an acute care facility or an inpatient rehabilitation facility, or if they refuse it. During the COVID-19 pandemic, an assessment of exercise capacity, performed at the sports medicine unit of the Province of Liège at three months (M3) after ICU discharge, was added to the standardized follow-up for patients who attended the M3 consultation at our follow-up clinic. Furthermore, the patients who attended this sports medicine consultation were prospectively recruited for an oxidative stress assessment after its approval by the same ethics committee (local reference 2020/227, 10 September 2020). Informed consent was obtained from the patients before blood collection.

#### 2.1.2. Patients with Long COVID after a Mild Infection (MLC Group)

Since the beginning of 2021, the sports medicine unit of the Province of Liège also increasingly assessed several patients complaining of persistent fatigue and exercise intolerance after a mild COVID-19 infection that did not require hospitalization. All patients who underwent a CPET in this context were included in our study.

#### 2.1.3. Obese Patients (OB Group)

Finally, obese patients with a BMI ≥ 30 kg/m^2^ (according to the World Health Organization’s classification for obesity based on BMI [15]) who underwent a CPET evaluation in the context of dyspnea at the University Hospital of Liège during the same period were retrospectively screened as a control group. We excluded obese patients with altered pulmonary function tests (defined as pulmonary flow volumes <80% predicted values); known interstitial lung disease; known ischemic, valvular, or rhythmic cardiopathy; and a recent history of moderate to severe COVID-19. The physical performances, demographics, and clinical data of these two groups were collected retrospectively and extracted from the medical files.

### 2.2. Cardiopulmonary Exercise Testing (CPET)

Patients underwent a symptom-limited, incremental CPET on a cycle ergometer (Lode Corival, CPET 960900, Groningen, the Netherlands at the sports medicine unit of the Province of Liège, and eBike ergometer, GE Healthcare, Wauwatosa, USA at the University Hospital of Liège) under the supervision of a trained sports physician. Patients were asked to continue their usual medications before the test. The protocol was similar in both sites and consisted of a 3 min warm-up with a fixed workload (30% of the predicted maximum workload). A personalized ramp increment in workloads of 15–25 watts every 1–2 min was then started and continued until exhaustion, which was defined as shortness of breath and/or leg fatigue. This was followed by a 3 min recovery. The ventilation and gas exchange variables were measured using a metabolic cart (Schiller Cardiovit CS-200 Excellence, Schiller AG, Baar, Switzerland, at the sports medicine unit of the Province of Liège, and Vyntus CPX, Vyaire Medical, Chicago, IL, USA, at the University Hospital of Liège). The calibrations were realized according to the manufacturer’s instructions.

The pulmonary flow volumes, including a measurement of the forced expiratory volume in 1 s (FEV1), were determined before CPET by spirometry in a sitting position, as recommended by the ATS/ERS guidelines [19]. The CPET was performed and interpreted based on ATS/ACCP guidelines [20]. Oxygen uptake (VO_2_), carbon dioxide output (VCO_2_), and minute ventilation (VE) were measured breath by breath during CPET. One metabolic equivalent (MET) was the resting oxygen uptake in a sitting position and equaled 3.5 mL/kg/min. The ventilatory equivalent for CO2 (Veq CO_2_) assessed the ventilatory efficiency (=VE/VCO_2_). At rest, the Veq CO_2_ was typically between 25 and 30. The adaptative threshold (ADT) was detected when the ventilation and cardiac adaptations to effort started. The anaerobic threshold (AT) was defined as the highest VO_2_ attained without a sustained increase in the blood lactate concentration and lactate–pyruvate ratio. It was detected metabolically as the point of inflection at which the VCO_2_ and VE increased relative to VO_2_. AT occurs typically between 47% and 64% of the peak VO_2_ in healthy untrained individuals. The respiratory exchange ratio (RER) was defined as RER = VCO_2_/VO_2_. A RER of 1 indicated a metabolism using primarily carbohydrates, whereas a RER < 1 resulted from a metabolism using a mixture of carbohydrates with fat (RER ~ 0.7) or protein (RER ~ 0.8). The heart rate (HR), 12-lead electrocardiogram (ECG), non-invasive blood pressure (BP), and pulse oximetry were monitored throughout. The oxygen pulse (πO_2_), a surrogate of stroke volume, was calculated by dividing VO_2_ by the heart rate, and it is typically around 5 mL/beat in healthy non-athletes.

The maximum predicted VO_2_ (a measurement of the maximal aerobic capacity) was calculated using the Wasserman equation. The maximum predicted heart rate was calculated using the Astrand formula: HR max predicted = 220 − age (years). The breathing reserve (BR) at maximum exercise was calculated as the maximum voluntary ventilation (MVV) minus the ventilation at the maximum of exercise (peak VE), and the result was divided by MVV ([MVV − peak VE]/MVV). In this protocol, the MVV was calculated by the multiplication of FEV1 value × 30. BR refers to how closely VE approaches MVV during exercise and is typically ≥20% (between 30 and 50%) in healthy non-athletes. The VE/VCO_2_ slope was also calculated and is typically <30–32 in healthy non-athletes. The chronotropic response (CR) to exercise was evaluated by the percentage of chronotropic reserve (% chronotropic reserve = [peak HR − resting HR/220 − age − resting HR] × 100). It is typically >85% in healthy non-athletes. The metabolic efficiency was calculated as the workload (converted in mL O_2_/min) divided by the peak VO_2_, and it is typically between 15% and 35% in healthy non-athletes.

The baseline data were recorded during the resting period. The peak data were recorded during the last 20 s of the test. The normal value for peak VO_2_ is >84% of the maximum predicted VO_2_. The peak πO_2_ is typically >80% of the maximum predicted πO_2_. The T1/2, i.e., the time required for a 50% decrease in VO_2_ from its peak value, was also recorded: it typically occurs 80 s after the end of effort in healthy non-athletes.

### 2.3. Biological Parameters of Critically Ill Survivors at M3

The biological data were generated from one single laboratory (Unilab, University Hospital of Liège, Liège, Belgium), ISO 15189 accredited. 

The following biomarkers related to inflammation and endocrine status were recorded: serum C-reactive protein (CRP), serum thyroid-stimulating hormone (TSH), thyroxine (T4), and serum cortisol (immunoassays, Abbott Alinity instrument, Lake Bluff, IL, USA). These analyses are part of our routine follow-up. In this context, blood samples were collected in the early afternoon.

The oxidative stress was investigated using the following method. Blood was collected 2 h before exercise testing, after a fasting period of at least 8 h. The blood samples that were drawn by venous punction on tubes containing EDTA and citrate were immediately centrifuged at 3000× *g* during 10 min. The serum was allowed to clot for 30 min before being centrifuged. The plasma and serum samples were then frozen at −80 °C until the analysis of the OS biomarkers. We then performed the blood determination of antioxidants, specifically vitamin C; thiol proteins (PSH); glutathione peroxidase (GPx); trace elements (copper (Cu), zinc (Zn), and the copper–zinc ratio); and the biomarkers of oxidative damages to lipids (lipid peroxides (ROOH), as previously described [21,22,23]. Myeloperoxidase (MPO) was assessed using an MPO ELISA kit (Immun Diagnostik, Bensheim, Germany). Albumin was assessed by spectrophotometry using an Alinity C kit (Abbott, Chicago, Lake Bluff, IL, USA). Hemoglobin, white blood cells, and neutrophils were determined by flow cytometry on Sysmex-XN device (Mississauga, ON, Canada)). 

### 2.4. Statistical Analyses

Statistical analysis was performed using the Graphpad Prism (version 9.0 for Mac OSX, Graphpad Inc., San Diego, CA, USA). The normality was assessed using the Shapiro–Wilk test. As some datasets did not pass the normality test, the results were expressed as medians with lower and upper quartiles [Q1–Q3] for quantitative parameters. The qualitative variables were described using count and percent. Comparisons between data were made using the Kruskal–Wallis test with Dunn’s post-hoc test. A chi-squared test was used to compare categorical variables. A *p* value < 0.05 was considered statistically significant.

## 3. Results

### 3.1. Patients’ Flow

From 1 March 2020 until 31 May 2021, 118 patients with COVID-19 ARDS survived an ICU stay ≥7 days in our hospital, of whom 68 patients attended our post-ICU follow-up clinic three months after ICU discharge (M3). The data from 31 of these patients were also explored at the sports medicine consultation using CPET (ICU group). They were compared with the 23 patients with symptoms of long COVID who attended the sports medicine consultation 12 [5–14] months after infection between February 2021 and February 2022 (MLC group). They benefited from a complete clinical assessment before CPET, and none of them presented cardiopulmonary sequelae of the COVID-19 infection. Finally, 15 obese patients met the inclusion criteria and underwent a CPET in the University Hospital of Liège (Belgium) during the same period. They were used as the control group (OB group). 

### 3.2. Patients’ Characteristics

The characteristics of the three groups of patients are detailed in Table 1. 

The spirometry data are depicted in Table 2: forced vital capacity (FVC), forced expiratory volume in 1 s (FEV1), and diffusion capacity of carbon monoxide (DLCO) were considered within normal values in the three groups.

### 3.3. CPET and Biological Correlates in the ICU Group

The CPET data at rest, during effort, and recovery are presented in Table 3. At rest, the VO_2_ and MET were increased compared with the normal ranges in healthy people. The RER was also slightly increased. The Veq CO_2_ was into normal values. 

Physiologic adaptations to effort (ADT) started early, after 3 [2–3.4] min, for a workload of 40.2 [33–60]% of the maximal predicted workload. All patients reported to have performed a maximal volitional effort up to their limits, but the effort was stopped before the maximal predicted workload and maximal predicted VO_2_ were reached. The AT was observed at high values of VO_2_.

The maximum effort was reached at low values of the workload and VO_2_. The peak Veq CO_2_ did not significantly increase during exercise compared with the resting state (*p* = 0.326). The VE/VCO_2_ slope was slightly higher than normal values. πO_2_ was significantly increased with effort compared with the resting state (*p* < 0.001). The CR was below normal values. On the contrary, the BR was considered normal. The metabolic efficiency was quite low: 15.2 [12.9–17.8]%. Despite the severe deconditioning observed, no adverse events were noticed during the CPET examination. 

During recovery, T1/2 was reached later than predicted: 130 [120–170] s after the end of the effort. The RER as well as the Veq CO_2_ were still increased at T1/2, while πO_2_ was decreased. Four minutes after the end of the effort, the QTc interval on the EKG was 422 [383–445] ms. 

The biological data are presented in Table 4. The thyroid tests and cortisol serum concentration were into normal ranges. Oxidative stress was assessed in 10 patients in the ICU group. The vitamin C concentration was below normal ranges in five (50%) patients. The PSH was decreased, while the GPx and Cu–Zn ratio were increased. The ROOH was elevated in five (50%) patients. While the CRP concentration was into normal ranges, the MPO concentration was slightly increased.

### 3.4. Comparison of CPET Data between the ICU Group and the MLC Group

The results of the comparison are detailed in Table 3. At rest, the VO_2_ and MET in the MLC group were at greater than normal values. However, compared with the ICU group, the VO_2_ and MET were similar in both groups. The Veq CO_2_ was higher than normal values and significantly higher than in the ICU group (*p* = 0.004). 

ADT occurred after 4 [3.4–4.2] min, which was later than in the ICU group (*p* < 0.001). At that time, the VO_2_ had already reached higher values than normally expected (52 [48–59]% peak VO_2_). As in the ICU group, the AT was observed at high values of VO_2._

The maximum effort was reached at higher values than the predicted maximal workload and VO_2_. The peak Veq CO_2_ tended to decrease during exercise compared with the resting state (*p* = 0.45), while πO_2_ significantly increased at effort compared with the resting state (*p* = 0.0002). The CR remained normal, but the BR was low. The VE/VCO_2_ slope was into normal ranges and did not differ from the ICU group. The metabolic efficiency was altered, reaching only 22 [21.5–23.5]%. However, the metabolic efficiency in the MLC group was higher than it was in the ICU group (*p* < 0.001). 

The recovery was delayed but occurred quicker in the MLC group compared with the ICU group. The RER came back to values below 1 and was lower than it was in the ICU group (*p* = 0.0007). The Veq CO_2_ reached 133.1 [121.7–144.7]% of peak Veq CO_2_, which was higher than it was in the ICU group (*p* < 0.001), in link with the bigger effort provided. Four minutes after the end of the effort, the QTc interval on the EKG was similar to the QTc interval in the ICU group: 394 [384–423] msec (*p* = 0.141).

### 3.5. Comparison of CPET Data between ICU Group and OB Group

The comparison between the two groups is detailed in Table 3. At rest, the VO_2_ and MET were lower in the OB group than they were in the ICU group (both *p* < 0.001). The Veq CO_2_ was at the upper limit of normal values, but it was not statistically different from the ICU group. 

The AT was observed at significantly lower VO_2_ in the OB group than it was in the ICU group (*p* < 0.001). 

The maximum effort was reached at a lower workload than the maximum predicted, but it was significantly higher than it was in the ICU group (*p* = 0.0003). However, the VO_2_ peak, expressed as a percentage of the maximal predicted VO_2_, was similar to the ICU group. Compared with the ICU group, the CR and BR were not different in the OB group. The VE/VCO_2_ slope was into normal ranges and did not differ from the ICU group. The metabolic efficiency was normal, reaching 27.5 [22.6–29.2]%, which was significantly higher than it was in the ICU group (*p* < 0.001).

Recovery occurred quicker in the OB group than it did in the ICU group (*p* = 0.004). The RER was significantly higher than it was in the ICU group, reaching 1.38 [1.29–1.45] (*p* < 0.001). The Veq CO_2_ and πO_2_ were similar in the two groups. 

## 4. Discussion

Using CPET, our pilot study, which included long COVID patients and obese patients as controls, provides a more comprehensive insight into the pathophysiology of exercise limitations in critical COVID-19 survivors. 

Our study confirms previous findings and demonstrates a reduced exercise capacity in critical COVID-19 survivors, associated with a slow and incomplete recovery and without signs of cardiac or ventilatory failure. The cardiac response to exercise seemed appropriate with a normal πO_2_ profile and suitable chronotropic adaptation, although most of these patients were treated with selective beta blockers. Moreover, the profile of the Veq CO_2_ was normal, potentially excluding any right ventricular dysfunction that could have persisted after ARDS. Importantly, significant metabolic alterations were also observed. At rest, a hypermetabolic status was noticed (i.e., high baseline VO_2_ and MET) in the absence of potential causes such as acute infection or endocrine abnormalities. Proteins were used as metabolic fuel, rather than lipids, as suggested by the elevated baseline RER. The oxygen consumption during exercise was disproportionate since three quarters of the maximum predicted VO_2_ was necessary in response to a peak workload of approximately two-thirds of the maximum predicted workload. The metabolic efficiency was dramatically low. Finally, post-exercise recovery was characterized by a persistent anaerobic metabolism. This resulted in lower metabolic reserves and earlier physiological adaptations to effort at high values of VO_2_. A slight hyperventilation was observed, resulting from the low metabolic efficiency. Altogether, the results of the CPET suggest that the observed exercise limitation cannot be explained by insufficient oxygen delivery secondary to persistent impairment of pulmonary or cardiac function, but rather by a metabolic disorder. Indeed, these patients presented signs of sustained hypermetabolism and impaired oxygen utilization. 

A potential explanation for the observed metabolic pattern is a persistent inflammation. Inflammation and neuroendocrine stress induce hypermetabolism after critical illness, resulting in numerous pathophysiologic alterations, including supraphysiologic metabolic rates, proteolysis, lipolysis, insulin resistance, gluconeogenesis, and futile substrate cycling [17,24]. Moreover, the body fails to recognize fat as source of energy and rather uses proteins as major fuel, leading to muscle protein breakdown and loss of muscle mass, due to the use of proteins as the primary fuel [18]. Inflammation has also been linked to mitochondrial dysfunction, potentially explaining the failure we observed in oxygen utilization. Inflammation persists in patients with a chronic critical illness [25], in burn patients several months after injury [26], and in ICU survivors at least 3 months following discharge [27]. In these patients, persistent inflammation has been associated with poor physical recovery. In our ICU group, we assessed systemic inflammation and oxidative stress using plasma MPO measurement. MPO is a pro-oxidant and cytotoxic enzyme from primary-granule polymorphonuclear neutrophils. We further evidenced that plasma MPO levels may remain elevated for at least 3 months after ICU discharge. In parallel, we also observed biological signs of increased oxidative stress and antioxidant defenses collapse. ROOH, an indicator of oxidative damage to lipids, was at the upper limit of the normal ranges and was substantially increased in 50% of the ICU patients. Reduced GPx (antioxidant enzyme) and an increased Cu–Zn ratio are indicators of an adaptative response to oxidative stress. On the contrary, depletion in PSH and vitamin C levels at the lower limit of the normal ranges are indicators of an antioxidant defense collapse. In a large number of pathologies, inflammation and oxidative stress have been shown to be closely related, one process being easily induced by the other [28]. Altogether, our data suggest that metabolic changes and muscle dysfunction in ICU survivors could result from protracted inflammation and oxidative stress. This persistent inflammation in ICU survivors is poorly understood. This phenomenon could be associated with tissue damages and their subsequent repair [29]. Stressors such as physical exercise can further increase the release of MPO [30], potentially to the point of exacerbating muscle dysfunction and blunt adaptation to exercise [31,32]. Whether physical rehabilitation and muscle exercises following ICU discharge could induce systemic and/or muscle inflammation is unknown.

Interestingly, patients with long COVID after a mild disease (MLC group) presented a similar metabolic profile to a lesser extent: hypermetabolism at rest, cardiopulmonary adaptation to effort occurring early at high percentages of peak VO_2_, low metabolic efficiency, and a slow and incomplete recovery. As in the ICU group, the cardiac response was normal. In the MLC group, hyperventilation was observed at rest but not at peak effort or during recovery, considering the generated effort that was significantly higher than it was in the other two groups. The respiratory response to exercise was considered adequate: in particular, the VE/VCO_2_ slope was considered normal. Similar findings have been described in other reports 3 months after COVID [33] or even later [9]. To date, such a hyperventilation pattern at rest is not fully understood. However, a relationship between the observed hypermetabolism and the observed hyperventilation at rest could be hypothesized for patients with a higher daily level of activities compared with ICU survivors. Unfortunately, we did not have the opportunity to assess the status of the inflammation and oxidative stress in these long COVID patients due to the retrospective design of the study. However, elevated interleukine-6 blood levels were recently reported in long COVID patients experiencing very severe impairments, at least in the domain of physical health, compared with patients with a better recovery [29].

The patients of the ICU group were all obese. The potential contribution of obesity on exercise limitation was thus questioned. In our OB group, exercise limitation was not associated with hypermetabolism at rest, AT occurred at normal percentages of peak VO_2_, and the metabolic efficiency was considered normal. According to our observations, the exercise limitation in obese patients was secondary to a deficit in the supply of oxygen to muscles, probably due to a pulmonary restrictive syndrome as suggested by an extreme anaerobic metabolism with a high RER during recovery. Altogether, these findings indicate that obesity is not a key contributor of exercise limitation in critical COVID-19 survivors.

Some limitations need to be acknowledged. First, the three cohorts of patients were limited, and the retrospective method could be responsible for a selection bias. However, our observations are in line with other published datasets. Second, this study lacks the precise assessment of pre-COVID exercise capacity. This is a common issue with many studies assessing long-term outcomes in ICU survivors or after infectious diseases and is related to the unpredictability of these conditions. This pitfall can lead to misinterpretation of what is considered as sequelae. However, all included patients were fully active before their COVID-19 infection. Finally, in the MLC group, some parameters that could have been useful for understanding their exercise profile (such as arterial blood gas chemistry or oxidative stress biomarkers) were not available.

## 5. Conclusions

Patients’ exercise capacity was dramatically reduced three months after a critical COVID-19 bout due to an altered metabolic profile rather than to cardiac or pulmonary residual impairments. This was associated with a persistent inflammation and oxidative stress, potentially explaining the depicted metabolic alterations. Altered exercise and metabolic profiles were also observed in long COVID patients, suggesting a common inflammatory mechanism. On the contrary, obese patients did not share the same dysmetabolic pattern as COVID-19 survivors, suggesting that obesity plays a minor role in the exercise limitation of COVID-19 ICU survivors. The results of our pilot study should be confirmed in larger cohorts and in non-COVID critically ill survivors. Further studies are also required to investigate how the mitigation of inflammation and oxidative stress could improve exercise capacity after critical illness and/or COVID-19 infection.

## Figures and Tables

**Table 1:** Characteristics of the three groups of patients.

Data		ICU Group *n* = 31	MLC Group *n* = 23	OB Group *n* = 15
Age, y		61 [54–67]	44 [37–50]	53 [45–69]
Male, n (%)		21 (67.7)	7 (30.4)	8 (53.3)
Weight, kg		96.1 [88.9–100]	76.3 [64.3–90.6]	94 [90–110]
BMI, kg/m^2^		32.9 [30.1–34.8]	25.8 [22.3–30]	33 [30.7–35.4]
Comorbidities	Diabetes	18 (58.1)	2 (8.7)	2 (13.3)
	Hypertension	18 (58.1)	3 (13)	7 (46.7)
	Cardiac disease †	9 (29)	0	2 (13.3)
	Respiratory disease ††	5 (16.1)	7 (30.4)	15 (100)
	Chronic kidney disease	1 (3.2)	0	0
	Active smoking	1 (3.2)	0	1 (6.7)
SOFA at ICU admission		5.5 [3.7–7]		
Mechanical ventilation, *n* (%)		22 (71)		
Duration of mechanical ventilation, d		15.5 [11.8–24]		
Corticosteroids, *n* (%)		22 (71)		
Renal replacement therapy, *n* (%)		3 (9.7)		
Extracorporeal membrane oxygenation, *n* (%)		0		
ICU LOS, d		15.4 [9.7–25.6]		
Hospital LOS, d		29 [21–42.7]		
Beta-blockers medication at CPET time		20 (64.5)	1 (4.3)	4 (26.7)

Data are expressed as the median with lower and upper quartiles [Q1–Q3]. BMI: body mass index; ICU: intensive care unit; LOS: length of stay; SOFA: sequential organ failure assessment. † includes ischemic heart disease, valvular disease, cardiomyopathies, and chronic heart disease. †† includes asthma, chronic obstructive pulmonary disease, and interstitial lung diseases.

**Table 2:** Pre-CPET spirometry in the three groups of patients.

Data	ICU Group *n* = 31	MLC Group *n* = 23	OB Group *n* = 15
FVC, % predicted	90 [72–104.5]	100 [95–110.8]	98 [90–106.5]
FEV1, % predicted	92 [80–105.5]	97.5 [89.5–104.3]	102 [89–106]
DLCO, % predicted	98 [84.5–110]	95 [82.2–101.8]	89 [84–104.5]

Data are expressed as the median with lower and upper quartiles [Q1–Q3] or count (%). DLCO: diffusion capacity of carbon monoxide; FEV1: forced expiratory volume in one second; FVC: forced vital capacity.

**Table 3:** CPET data in the three groups of patients.

Data		ICU Group *n* = 31	MLC Group *n* = 23	Adjusted *p* Value (Comparison between ICU Group and LC Group)	OB Group *n* = 15	Adjusted *p* Value (Comparison between ICU Group and OB Group)	Kruskal–Wallis Test *p* Value
Maximum predicted	VO_2_ (mL/min/kg)	24.8 [19.7–28]	26.2 [23.7–33.4]	NS	22.3 [15.5–25.5]	NS	0.048
	HR (bpm)	159 [152–167]	176 [170–183]	<0.001	168 [151–190]	NS	0.001
	Workload (W)	155.5 [131–172]	136 [128–182]	NS	135 [103–185]	NS	NS
	πO_2_ (mL/beat)	15.8 [10.8–17.1]	10.3 [9.1–15.2]	NS	14.5 [11.7–18.8]	NS	0.036
Resting state	HR (bpm)	77 [66–87]	76 [66–86]	NS	91 [74–99]	NS	0.051
	Systolic blood pressure (mmHg)	140 [130–145]	120 [110–120]	<0.001	125 [117–130]	NS	<0.001
	Diastolic blood pressure (mmHg)	70 [70–70]	60 [60–70]	0.03	80 [70–85]	NS	0.004
	VO_2_ (mL/min/kg)	8 [5.6–9.7]	6.1 [4.4–7.6]	NS	4.4 [3.3–5.2]	<0.001	0.001
	MET	2.4 [1.8–3.1]	1.7 [1.3–2.3]	NS	1.3 [0.9–1.5]	<0.001	<0.001
	RER	0.85 [0.8–0.91]	0.77 [0.73–0.82]	0.001	0.8 [0.76–0.84]	NS	0.002
	πO_2_ (mL/beat)	7.7 [6.2–13.2]	5.5 [4.8–6.1]	0.028	4.1 [3.3–5.2]	<0.001	<0.001
	Veq CO_2_	29.6 [26.2–34.4]	35.9 [31.3–40.1]	0.004	32.3 [30.4–34.5]	NS	0.005
ADT	VO_2_ (% peak)	66 [57–74]	52 [48–59]	<0.001			
AT	VO_2_ (% peak)	81 [72.5–87.2]	85 [75–91]	NS	44.5 [34.5–58.3]	<0.001	<0.001
Peak	Workload (% max predicted)	66 [40.9–79.2]	104.4 [95.6–122.3]	<0.001	94.7 [77.7–123.9]	0.003	<0.001
	VO_2_ (% max predicted)	74.5 [62.6–102.8]	105.3 [86.8–132.8]	0.005	74 [62–84.3]	NS	<0.001
	HR (% max predicted)	76.7 [65.1–90.3]	97.9 [88.7–101.1]	<0.001	80.6 [74–89.3]	NS	<0.001
	πO_2_ (% max predicted)	109 [75.5–135.4]	116 [105–134]	NS	72.9 [56.4–85.4]	0.002	<0.001
	Veq CO_2_	29.5 [26.6–34.4]	33 [30.4–40.9]	NS	31.5 [29.6–34.3]	NS	0.117
	VE (l/min)	61.1 [44.7–72.8]	79.4 [64.4–89.5]	0.007	60 [40–71]	NS	0.004
	VE/VCO_2_ slope	33.6 [29.1–44.9]	26 [22.9–34.9]	NS	32.1 [29.5–33.5]	NS	0.066
	CR (%)	57.8 [35.5–79.3]	96.5 [73.2–101.7]	<0.001	59.5 [43.1–71.7]	NS	<0.001
	BR (%)	33 [19.8–41.5]	19.1 [6.6–31.2]	NS	48 [30–58]	NS	0.001
T1/2	VO_2_ (% peak)	51 [49.2–53]	50 [49–51]	NS	49.4 [45.2–52.4]	NS	0.212
	HR (% peak)	79.2 [75–85]	69.2 [64.6–74.2]	<0.001	78.7 [76.4–90.4]	NS	<0.001
	RER	1.13 [1–1.2]	0.97 [0.91–1.03]	0.021	1.38 [1.29–1.45]	<0.001	<0.001
	πO_2_ (% peak)	62.4 [56.2–69.9]	47.6 [42.2–53.8]	<0.001	59.7 [54.8–68.2]	NS	<0.001
	Veq CO_2_ (% peak)	107.4 [100.2–117.4]	133.1 [121.7–144.7]	<0.001	107.8 [102.9–114.5]	NS	<0.001

Data are expressed as the median with lower and upper quartiles [Q1–Q3] or count (%). NS: non-significant.

**Table 4:** Biological parameters in the ICU group.

Data	*n*	Blood Concentrations	Reference Ranges
C-reactive protein (CRP), mg/L	31	1.95 [0.95–2.69]	0–5
Thyroid-stimulating hormone (TSH), mUI/L	31	1.05 [0.49–1.75]	0.35–4.94
Thyroxine (T4), pmol/L	31	10.95 [9.82–13.23]	8.7–16.8
Cortisol, nmol/L	31	210.9 [152.4–267.8]	80–477.3
Albumin, g/L	10	44 [41–46.25]	35–52
Hemoglobin, g/dL	10	13 [10.93–14.7]	Male: 13.2–17.2 Female: 11.7–15
White blood cells, 10^3^/mm^3^	10	7.27 [6.2–8.32]	4.6–10.1
Neutrophils, %	10	54.6 [39.8–62.3]	42.2–71
Vitamin C, mg/L	10	7.32 [5.19–9.88]	6–18
Thiol proteins (PSH), µM	10	296 [260–360]	310–523
Glutathione peroxidase (GPx), UI/g	10	64.5 [61.7–90]	20–58
Cupper (Cu), mg/L	10	0.91 [0.82–1.19]	0.7–1.1
Zinc (Zn), mg/L	10	0.8 [ 0.73–0.99]	0.7–1.2
Cu/Zn ratio	10	1.18 [0.95–1.24]	1–1.17
Lipid peroxides (ROOH), µM	10	427 [366–949.3]	0–432
Myeloperoxidase (MPO), ng/mL	10	92 [75.5–106.5]	0–55

Data are expressed as the median with lower and upper quartiles [Q1–Q3].

## Data Availability

The datasets used and/or analyzed during the current study are available from the corresponding author upon reasonable request.
